# Invasive micropapillary carcinoma of the breast has a better long‐term survival than invasive ductal carcinoma of the breast in spite of its aggressive clinical presentations: a comparison based on large population database and case–control analysis

**DOI:** 10.1002/cam4.1227

**Published:** 2017-10-26

**Authors:** Hongliang Chen, Kejin Wu, Maoli Wang, Fuwen Wang, Mingdi Zhang, Peng Zhang

**Affiliations:** ^1^ Department of Breast Surgery Obstetrics and Gynecology Hospital of Fudan University Shanghai 200011 China

**Keywords:** Breast cancer‐specific survival, case–control analysis, invasive ductal carcinoma, invasive micropapillary carcinoma of the breast, overall survival

## Abstract

There are controversies in the comparison of overall survival between invasive micropapillary carcinoma of the breast (IMPC) and invasive ductal carcinoma (IDC). The objective of this study was to compare the long‐term survival outcome between non‐metastatic IMPC and IDC. The Surveillance, Epidemiology, and End Results database was searched to identify women with non‐metastatic IMPC and IDC diagnosed between 2001 and 2013. Comparisons of patient and tumor characteristics were performed using Pearson's chi‐square. The propensity score matching method was applied with each IMPC matched to one IDC. Breast cancer‐specific survival (BCSS) and overall survival (OS) were estimated using the Kaplan–Meier product limit method and compared across groups using the log‐rank statistic. Multivariate analysis was performed through Cox models. IMPC was presented with aggressive clinical presentations such as larger tumor, more positive lymph nodes, and more advanced stage compared with IDC. A higher rate of estrogen receptor (ER)/progesterone receptor (PR) positivity was also observed in IMPC. With a median follow‐up of 64 months, IMPC had a better BCSS (*P* = 0.031) and OS (*P* = 0.012) compared with IDC. In a case–control analysis IMPC was still an independent favorable prognostic factor for BCSS (HR = 0.410, *P* < 0.001, 95% CI: 0.293–0.572) and OS (HR = 0.497, *P* < 0.001, 95% CI: 0.387–0.637). In subgroup analysis, IMPC always showed a better survival outcome compared with IDC except in AJCC stage I and histologic grade I disease. IMPC has a better long‐term survival outcome compared with IDC in spite of its highly aggressive clinical presentation.

## Introduction

Invasive micropapillary carcinoma (IMPC) is a relatively rare histologic subtype of invasive breast cancer, accounting for approximately 1.0–8.4% of all breast cancer cases 1, 2, 3, 4, 5. It was first described by Siriaunkgul and Tavassoli in 1993 and listed in the 2003 World Health Organization (WHO) histologic classification of tumors of the breast as a subtype of invasive carcinoma 6, 7. This form of breast cancer, whether in its pure form or mixed with other types, is defined by its unique histology consisting of tight clusters of tumor cells, often arranged around central non‐vascular lumena, contained within cyst‐like spaces 8. Pathologically, it is known for its high proclivity for lymphovascular invasion (LVI), lymph node (LN) metastasis, exhibiting a more aggressive behavior than invasive ductal carcinomas (IDC) 7, 9. So, it is commonly presumed that IMPC has an unfavorable prognosis compared with IDC.

There has been some studies evaluating the prognosis of IMPC 3, 4, 10, 11, 12, 13, 14, 15, 16, and some controversies still exist in the comparison of breast cancer‐specific survival (BCSS) and overall survival (OS) outcome between IMPC and IDC, probably due to the low incidence of IMPC and inadequate follow‐up. Among the previous studies, Chen et al. declared a similar BCSS and OS between IMPC and IDC based on cases between 2001 and 2008 from the Surveillance, Epidemiology, and End Results (SEER) database 3, 4. The study above included cases with metastatic disease as well as early stage breast cancer. Moreover, the study did not include the information of chemotherapy. It was an important factor associated with the prognosis of IMPC, which exhibited highly aggressive clinical characterization.

So we further conducted a retrospective study which enrolled only non‐metastatic cases diagnosed between 2001 and 2013 with a longer follow‐up to compare the long‐term survival outcome between IMPC and IDC based on SEER 18 database and in addition a case–control analysis to balance the effects of baseline differences.

## Materials and Methods

### Patient population

This retrospective study employed data derived from the National Cancer Institute's limited use SEER 18 registry databases that were released in November 2016. We identified IMPC cases with International Classification of Disease for Oncology, third edition (ICD‐O‐3) code of 8507 and IDC cases with ICD‐O‐3 code of 8500. Search criteria were restricted to patients who were female and had histologically confirmed invasive carcinoma. Patients with more than one primary cancer, having metastatic disease at diagnosis, diagnosed at death or autopsy only, Tis or T0 stage, unknown T stage or N stage or estrogen receptor (ER)/progesterone receptor (PR) status, or patients without surgery were excluded. Because IMPC was first recorded in the SEER in 2001, we selected IMPC and IDC cases diagnosed between 1 January 2001 and 31 December 2013. SEER 18 provided an adjusted AJCC 6th edition criteria for cases diagnosed between 1998 and 2003, a derived AJCC 6th edition criteria for cases between 2004 and 2009, and a derived AJCC 7th edition criteria for cases between 2010 and 2013. Borderline ER or PR status was considered positive as ER/PR positivity was defined as >1% positive now. Poorly differentiated and anaplastic histologic grades were considered grade III disease.

We obtained permission to access the files of SEER program custom data with additional treatment fields such as radiation therapy and chemotherapy. The informed consent was not required because personal identifying information was not involved. This study was reviewed and approved by the Institutional Review Board of Obstetrics and Gynecology Hospital of Fudan University.

### Statistical analysis

Comparisons of the characteristics between IMPC and IDC were performed using Pearson's chi‐square test. Follow‐up cutoff was 31 December 2014. OS was computed from the time of diagnosis of breast cancer to the time of death from any cause or last follow‐up with patients still alive at last follow‐up censored. BCSS was computed from the time of diagnosis of breast cancer to the time of death from breast cancer with patients who died of other causes or still alive at last follow‐up censored. To diminish the effects of baseline differences in patients and clinical characteristics across histology subtypes for outcome differences, the propensity score matching method was applied with each IMPC patient matched to one IDC patient who showed similar characteristics in terms of year of diagnosis, histologic grade, tumor size stage, node stage, ER status, and so on. Survival outcomes were estimated using the Kaplan–Meier product limit method and compared across groups using the log‐rank statistic. Adjusted hazard ratios (HRs) with 95% confidence intervals were calculated using Cox proportional hazards model to assess the multivariable relationship of various patient and tumor characteristics and the survival outcomes. Two‐sided *P* < 0.05 was considered statistically significant. All the statistical analyses were performed using SPSS 22.0 software package (SPSS, Chicago, IL, USA).

## Results

### Clinical characteristics between IMPC and IDC

In all, 984 patients with IMPC and 317 478 patients with IDC were enrolled according to the inclusion criteria. The clinical characteristics of the whole cohort were summarized in Table 1.

**Table 1:** Comparison of clinical characteristics between IMPC and IDC

	IMPC (%)	IDC (%)	*P* value
Age			0.050
<60	496 (50.4)	169 936 (53.5)	
≥60	488 (49.6)	147 542 (46.5)	
Race			0.010
White	750 (76.2)	254 378 (80.1)	
Black	130 (13.2)	32 900 (10.4)	
Asian or Indian	97 (9.9)	28 669 (9.0)	
Unknown	7 (0.7)	1531 (0.5)	
Marital status			0.035
Married	546 (55.5)	183 940 (57.9)	
Unmarried	388 (39.4)	121 930 (38.4)	
Unknown	50 (5.1)	11 608 (3.7)	
Grade			0.000
Grade I	72 (7.3)	59 617 (18.8)	
Grade II	516 (52.4)	126 152 (39.7)	
Grade III	363 (36.9)	123 643 (39.0)	
Unknown	33 (3.4)	8066 (2.5)	
AJCC stage			0.000
I	372 (37.8)	163 128 (51.4)	
II	390 (39.6)	115 677 (36.4)	
III	222 (22.6)	38 673 (12.2)	
T stage			0.000
T1	575 (58.4)	201 710 (63.5)	
T2	308 (31.3)	95 109 (30.0)	
T3	77 (7.8)	13 290 (4.2)	
T4	24 (2.4)	7369 (2.3)	
N stage			0.000
N0	479 (48.7)	212 494 (66.9)	
N1	310 (31.5)	75 494 (23.8)	
N2	106 (10.8)	19 945 (6.3)	
N3	89 (9.0)	9545 (3.0)	
ER			0.000
Negative	118 (12.0)	71 606 (22.6)	
Positive	866 (88.0)	245 872 (77.4)	
PR			0.000
Negative	239 (24.3)	104 319 (32.9)	
Positive	745 (75.7)	213 159 (67.1)	
Breast surgery			0.000
BCS	532 (54.1)	190 216 (59.9)	
Reconstruction	135 (13.7)	29 091 (9.1)	
CPM	94 (9.6)	25 822 (8.1)	
Mastectomy	278 (28.3)	85 757 (27.0)	
Lymph nodes removed			0.000
None	46 (4.7)	14 115 (4.4)	
<10	574 (58.3)	205 762 (64.8)	
≥10	361 (36.7)	96 284 (30.3)	
Unknown	3 (0.3)	1317 (0.4)	
Radiation therapy			0.399
None or unknown	429 (43.6)	142 667 (44.9)	
Yes	555 (56.4)	174 811 (55.1)	
Chemotherapy			0.000
None or unknown	454 (46.1)	172 763 (54.4)	
Yes	530 (53.9)	144 715 (45.6)	

The median age of the cohort was 58 years old. IMPC had a more advanced tumor stage. When comparing staging at presentation, IMPC patients had more T3 and T4 (10.2% vs. 6.5%) tumors, a lower percentage of grade I tumors (7.3% vs. 18.8%), a higher percentage of N2 (10.8% vs. 6.3%) or N3 (9.0% vs. 3.0%) stage and AJCC III stage disease (22.6% vs. 12.2%) compared with IDC patients. IMPC was presented with higher rate of ER (88.0% vs. 77.4%) and PR positivity (75.7% vs. 67.1%) compared with IDC patients.

Regional lymph nodes were examined in 942 IMPC patients (95.7%), with a median of five lymph nodes examined. 58.3% of patients had less than 10 lymph nodes removed or lymph node biopsy, whereas 36.7% had at least 10 lymph nodes removed or lymph node dissection.

In the aspect of treatment procedures, IMPC had a lower rate of breast conserving surgery (54.1% vs. 59.9%) compared with IDC. But in the patients receiving mastectomy, a higher rate of breast reconstruction (13.7% vs. 9.1%) and contralateral prophylactic mastectomy (CPM) (9.6% vs. 8.1%) was observed in IMPC. More patients with IMPC had at least 10 lymph nodes removed (36.7% vs. 30.3%). A higher percentage of chemotherapy was observed in IMPC (53.9% vs. 45.6%), but the percentage of radiation therapy for IMPC patients was similar to that in IDC patients.

### Comparison of long‐term survival outcome between IMPC and IDC

For the total study population, the median follow‐up was 64 months. At the time of last follow‐up, 99 (10.1%) patients with IMPC were dead, with 51 (5.2%) patients dead from disease; 50 572 (15.9%) patients with IDC were dead, with 26 347 (8.3%) patients dead from disease.

Among the clinical characteristics related to the prognosis, AJCC stage was the independent prognostic factors of IMPC for both BCSS (I vs. III: HR = 0.240, *P* < 0.001, 95% CI: 0.108–0.531; II vs. III: HR = 0.310, *P* = 0.001, 95% CI: 0.157–0.609) and OS (I vs. III: HR = 0.220, *P* < 0.001, 95% CI: 0.116–0.416; II vs. III: HR = 0.316, *P* < 0.001, 95% CI: 0.182–0.549). ER status was the independent prognostic factor of IMPC for BCSS (ER positive vs. negative: HR = 0.391, *P* = 0.030, 95% CI: 0.167–0.915).

**Figure 1:**
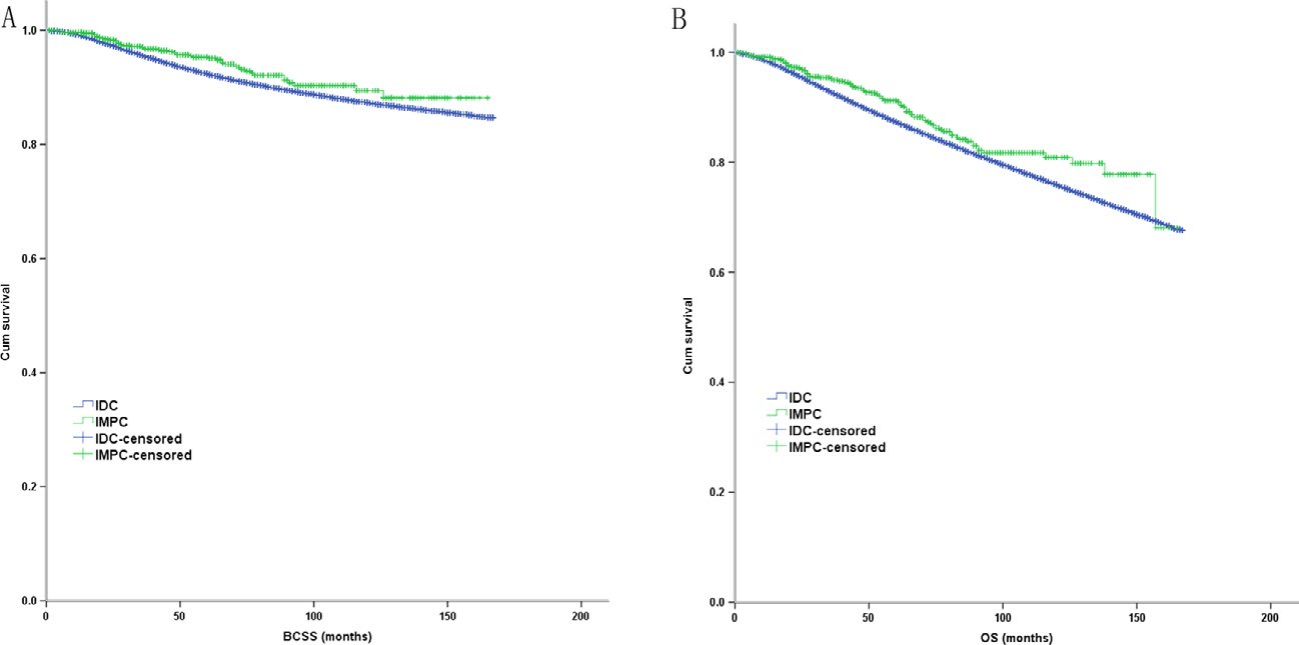
The Kaplan–Meier survival curves of BCSS and OS stratified by the histologic type of IMPC (*n* = 984) and IDC (*n* = 317 478) in the comparison based on large population database ((A): BCSS; (B) OS).

According to the comparison based on the large population database, IMPC had a better BCSS (*P* = 0.031) and OS (*P* = 0.012) compared with IDC (Fig. 1). In the multivariate COX analysis, IMPC histologic type was an independent favorable prognostic factor for BCSS (HR = 0.628, *P* = 0.001, 95% CI: 0.477–0.826) and OS (HR = 0.672, *P* < 0.001, 95% CI: 0.552–0.819).

**Figure 2:**
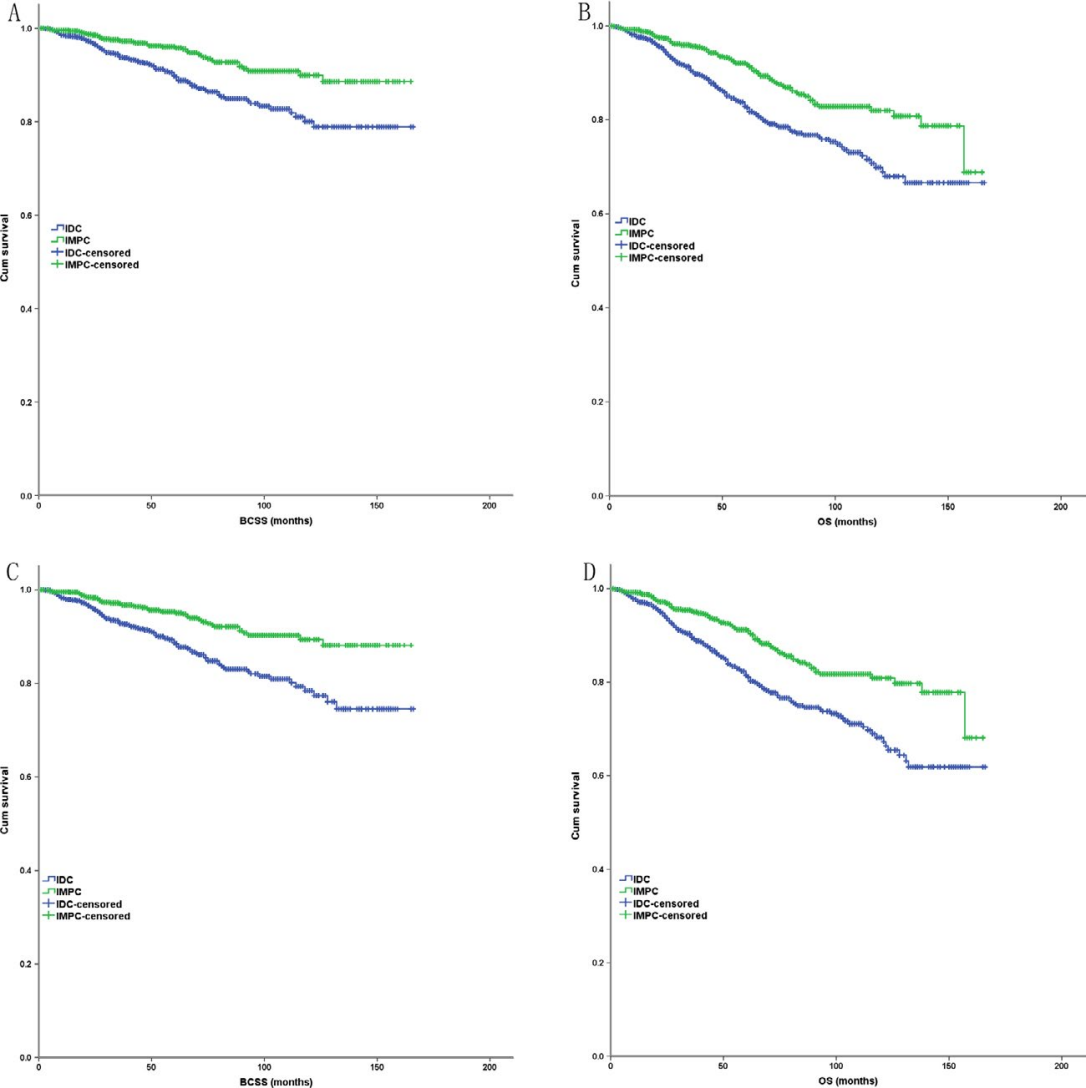
The Kaplan–Meier survival curves of BCSS and OS stratified by the histologic type of IMPC and IDC (*n* = 984 in each group) in the case–control analysis ((A) BCSS in completely matched case–control analysis; (B) OS in completely matched case–control analysis; (C) BCSS in partly matched case–control analysis; (D) OS in partly matched case–control analysis).

A 1:1 matched case–control analysis was conducted due to the great baseline difference between IMPC and IDC (Table 1). Cases were matched on the basis of year of diagnosis, age stage, histologic grade, tumor size stage, node stage, ER, PR and HER2 status, breast and lymph node surgery, radiation therapy, and chemotherapy. Only 916 out of 984 IMPC patients (93.1%) could be identified for completely matching in terms of all the variables above. In the matched groups, there was no significant difference in race (*P* = 0.291) and marital status (*P* = 0.126) between IMPC and IDC. According to the completely matched analysis, IMPC had a better BCSS and OS (*P* < 0.001) compared with IDC (Fig. 2A and B).

In all, 68 IMPCs lost in the completely matched study above, among which 39 cases (57.4%) belonged to AJCC stage III. To avoid the underlying biases, the 68 IMPC cases were further 1:1 matched to IDC for at least nine variables including year of diagnosis, histologic grade, tumor size stage, node stage, ER status, and so on (Table 2). Thus, all the IMPC cases together with matched IDC cases constituted a partly matched case–control analysis cohort. No significant difference in each characteristic was observed between IMPC and IDC in this cohort (Table 2). In this partly matched analysis, IMPC still had a better BCSS and OS (*P* < 0.001) compared with IDC (Fig. 2C and D). In the multivariate COX analysis, IMPC was still an independent favorable prognostic factor for BCSS (HR = 0.410, *P* < 0.001, 95% CI: 0.293–0.572) and OS (HR = 0.497, *P* < 0.001, 95% CI: 0.387–0.637) (Table 3).

**Table 2:** Comparison of clinical characteristics between IMPC and IDC in case‐matched analysis

	IMPC (%)	IDC (%)	*P* value
Age			0.964
<60	496 (50.4)	497 (50.5)	
≥60	488 (49.6)	487 (49.5)	
Race			0.161
White	750 (76.2)	791 (80.4)	
Black	130 (13.2)	104 (10.6)	
Asian or Indian	97 (9.9)	83 (8.4)	
Unknown	7 (0.7)	6 (0.6)	
Marital status			0.297
Married	546 (55.5)	515 (52.3)	
Unmarried	388 (39.4)	422 (42.9)	
Unknown	50 (5.1)	47 (4.8)	
Histologic grade			1.000
Grade I	72 (7.3)	72 (7.3)	
Grade II	516 (52.4)	516 (52.4)	
Grade III	363 (36.9)	363 (36.9)	
Unknown	33 (3.4)	33 (3.4)	
AJCC stage			1.000
I	372 (37.8)	372 (37.8)	
II	390 (39.6)	390 (39.6)	
III	222 (22.6)	222 (22.6)	
T stage			1.000
T1	575 (58.4)	575 (58.4)	
T2	308 (31.3)	308 (31.3)	
T3	77 (7.8)	77 (7.8)	
T4	24 (2.4)	24 (2.4)	
N stage			1.000
N0	479 (48.7)	479 (48.7)	
N1	310 (31.5)	310 (31.5)	
N2	106 (10.8)	106 (10.8)	
N3	89 (9.0)	89 (9.0)	
ER			1.000
Negative	118 (12.0)	118 (12.0)	
Positive	866 (88.0)	866 (88.0)	
PR			1.000
Negative	239 (24.3)	239 (24.3)	
Positive	745 (75.7)	745 (75.7)	
Breast surgery			1.000
BCS	532 (54.1)	532 (54.1)	
Mastectomy	452 (45.9)	452 (45.9)	
Lymph nodes removed			0.932
None	46 (4.7)	41 (4.2)	
<10	574 (58.3)	576 (58.5)	
≥10	361 (36.7)	363 (36.9)	
Unknown	3 (0.3)	4 (0.4)	
Radiation therapy			0.891
None or unknown	429 (43.6)	426 (43.3)	
Yes	555 (56.4)	558 (56.7)	
Chemotherapy			0.684
None or unknown	454 (46.1)	445 (45.2)	
Yes	530 (53.9)	539 (54.8)	

**Table 3:** Univariate and multivariate analysis of prognostic factors of the case‐matched analysis of IMP and IDC

	BCSS						OS					
	Univariate analysis			Multivariate analysis			Univariate analysis			Multivariate analysis		
	HR	*P*	95.0% CI	HR	*P*	95.0% CI	HR	*P*	95.0% CI	HR	*P*	95.0% CI
Age												
<60 versus ≥60	0.945	0.721	0.693–1.289				0.535	0.000	0.418–0.684	0.412	0.000	0.317–0.535
Race		0.002			0.011			0.000			0.001	
Black versus White	1.873	0.002	1.268–2.766	1.503	0.044	1.011–2.235	1.716	0.001	1.264–2.331	1.552	0.006	1.135–2.123
Asian or Indian versus White	0.470	0.052	0.219–1.008	0.388	0.016	0.180–0.837	0.424	0.006	0.231–0.777	0.421	0.006	0.228–0.776
Marital status												
Unmarried versus married	1.370	0.052	0.997–1.884				1.612	0.000	1.265–2.056	1.201	0.153	0.934–1.545
Grade		0.000			0.000			0.000			0.022	
I versus III	0.156	0.000	0.057–0.423	0.383	0.065	0.138–1.062	0.450	0.003	0.268–0.757	0.838	0.523	0.488–1.439
II versus III	0.356	0.000	0.255–0.497	0.538	0.000	0.380–0.761	0.578	0.000	0.451–0.741	0.772	0.055	0.593–1.005
Histologic type												
IMPC versus IDC	0.445	0.000	0.319–0.621	0.410	0.000	0.293–0.572	0.536	0.000	0.419–0.686	0.497	0.000	0.387–0.637
AJCC stage		0.000			0.000			0.000			0.000	
I versus III	0.087	0.000	0.051–0.146	0.083	0.000	0.042–0.161	0.222	0.001	0.161–0.306	0.153	0.000	0.099–0.238
II versus III	0.284	0.000	0.202–0.398	0.281	0.000	0.187–0.422	0.426	0.000	0.326–0.556	0.356	0.000	0.257–0.494
ER status												
Positive versus negative	0.322	0.000	0.231–0.450	0.410	0.000	0.248–0.677	0.465	0.000	0.353–0.614	0.611	0.013	0.414–0.902
PR status												
Positive versus negative	0.494	0.000	0.360–0.678	1.079	0.749	0.675–1.725	0.615	0.000	0.480–0.788	0.888	0.499	0.629–1.254
Lymph node removed		0.000			0.144			0.000			0.000	
None versus ≥10	0.637	0.250	0.296–1.373	2.681	0.020	1.165–6.172	1.717	0.009	1.145–2.575	3.402	0.000	2.082–5.557
<10 versus ≥10	0.389	0.000	0.281–0.540	1.188	0.391	0.801–1.760	0.475	0.000	0.369–0.613	1.010	0.951	0.741–1.376
Breast surgery												
BCS versus mastectomy	0.418	0.000	0.302–0.580	0.783	0.169	0.553–1.110	0.522	0.000	0.410–0.665	0.863	0.319	0.646–1.153
Radiation therapy												
Yes versus none or unknown	0.801	0.160	0.587–1.092				0.660	0.001	0.520–0.837	0.624	0.000	0.479–0.813
Chemotherapy												
Yes versus none or unknown	2.424	0.000	1.709–3.439	0.768	0.201	0.512–1.151	0.956	1.007	0.794–1.276			

**Figure 3:**
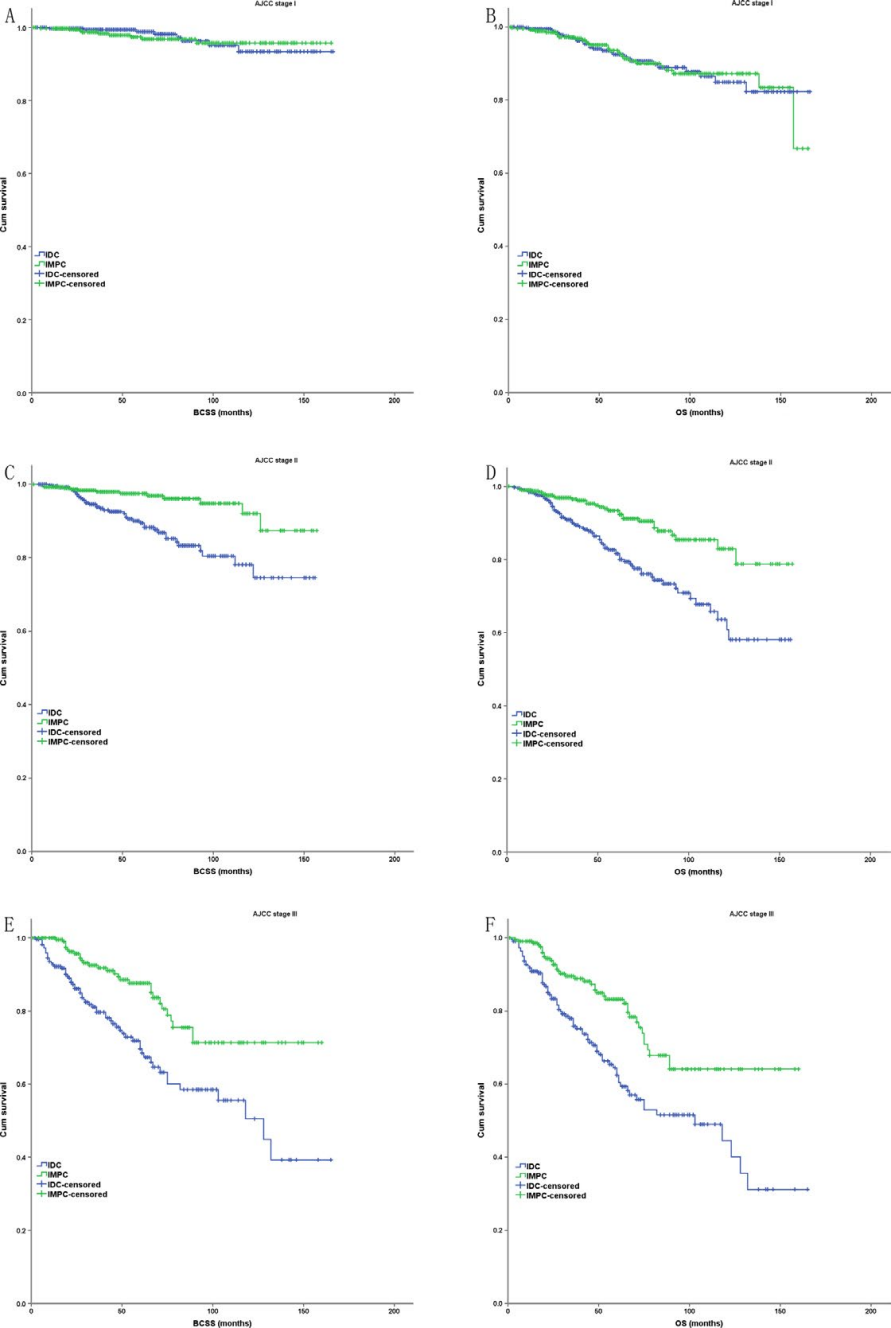
The Kaplan–Meier survival curves of BCSS and OS for the IMPC and IDC stratified by AJCC stage in case–control analysis ((A) BCSS in stage I; (B) OS in stage I; (C) BCSS in stage II; (D) OS in stage II; (E) BCSS in stage III; (F) OS in stage III).

**Figure 4:**
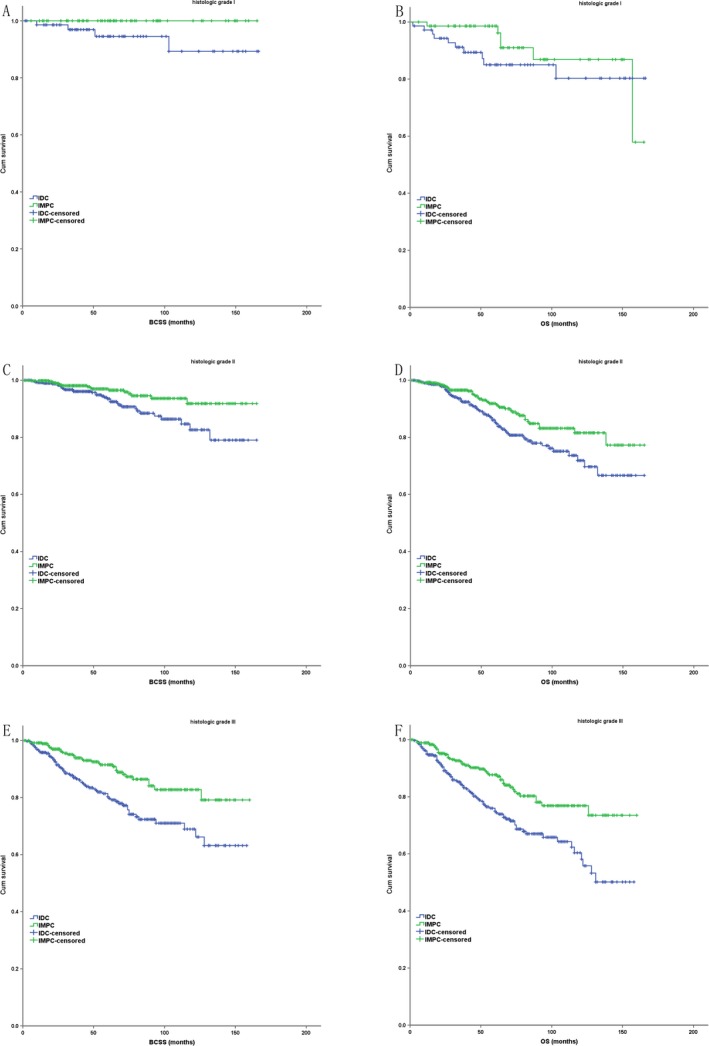
The Kaplan–Meier survival curves of BCSS and OS for the IMPC and IDC stratified by histologic grade in case–control analysis ((A) BCSS in grade I; (B) OS in grade I; (C) BCSS in grade II; (D) OS in grade II; (E) BCSS in grade III; (F) OS in grade III).

**Figure 5:**
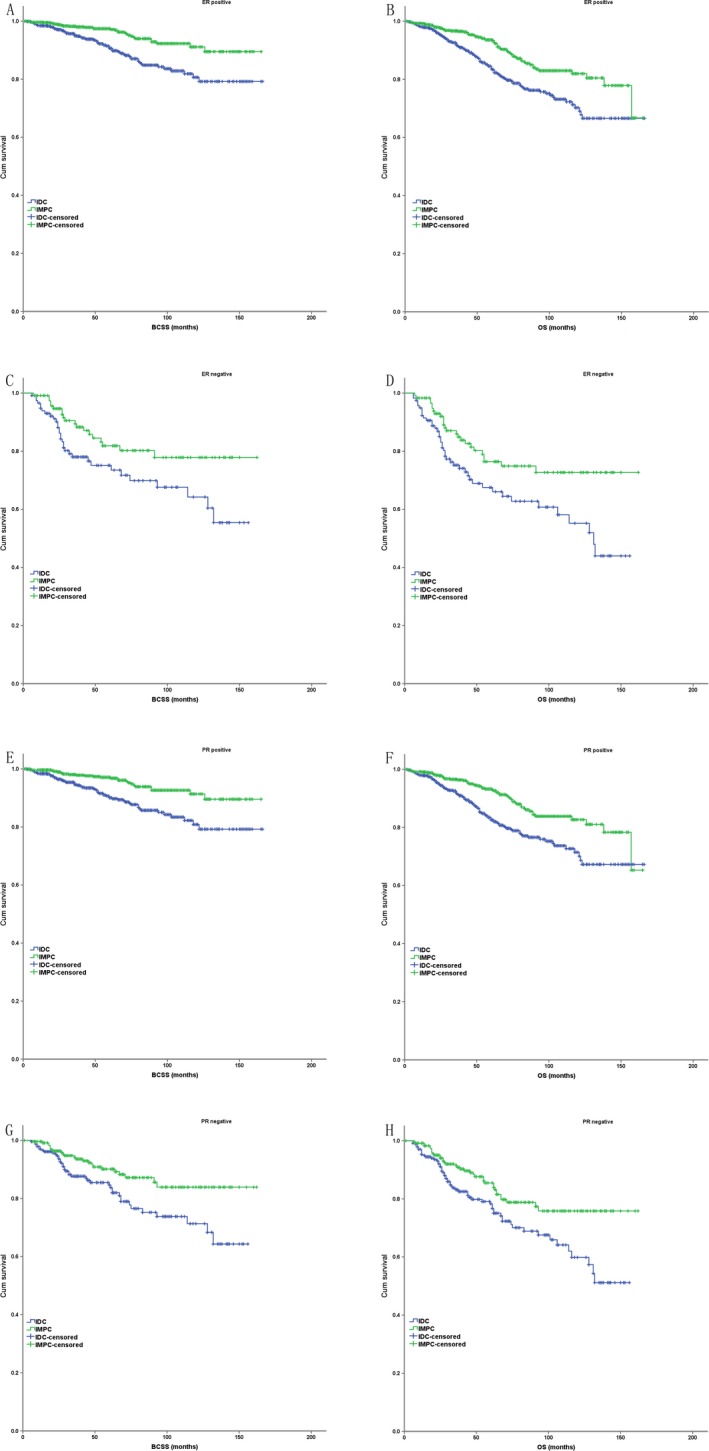
The Kaplan–Meier survival curves of BCSS and OS for the IMPC and IDC stratified by ER/PR status in case–control analysis ((A) BCSS in ER positive status; (B) OS in ER positive status; (C) BCSS in ER negative status; (D) OS in ER negative status; (E) BCSS in PR positive status; (F) OS in PR positive status; (G) BCSS in PR negative status; (H) OS in PR negative status).

In the subgroup analysis based on the partly matched study cohort, IMPC had a better BCSS and OS compared with IDC in AJCC stage II and III (*P* < 0.001 for BCSS and OS), but had a similar BCSS and OS with IDC in AJCC stage I (*P* = 0.803 for BCSS, *P* = 0.976 for OS) (Fig. 3). IMPC had a better BCSS and OS compared with IDC in histologic grade II (*P* = 0.007 for BCSS, *P* < 0.001 for OS) and III (*P* < 0.001 for BCSS, *P* < 0.001 for OS), but had a similar BCSS and OS with IDC in histologic grade I (*P* = 0.005 for BCSS, *P* = 0.266 for OS) (Fig. 4). IMPC had a better BCSS and OS compared with IDC in each ER status (ER positive: *P* < 0.001 for BCSS and OS; ER negative: *P* = 0.029 for BCSS, *P* = 0.012 for OS) and PR status (PR positive: *P* < 0.001 for BCSS and OS; ER negative: *P* = 0.009 for BCSS, *P* = 0.008 for OS) (Fig. 5).

## Discussion

IMPC is a rare histologic type that appears to be more aggressive compared with other breast cancers due to its increased probability of lymphovascular invasion and lymph node metastasis 10, 12. But there is still controversy with regard to the prognosis of IMPC compared with IDC. As far as our knowledge, our study is the first to confirm that IMPC has a better long‐term overall survival than IDC, which has the largest sample size by far with a relatively long follow‐up period for both large population database study and the further case–control study.

IMPC exhibited a higher rate of poor prognostic factors such as larger tumor size, a greater proportion of nodal involvement, a greater number of positive lymph nodes, and an increased incidence of LVI compared with IDC, which was related to prognosis 9, 10, 17, 18. In our study, the percentage of lymph node metastasis (51.3%), tumor larger than 2 cm (41.6%) and stage III disease (22.6%) were significantly higher than those of IDC, resulting in higher percentage of chemotherapy. But in previous studies, the incidence of lymph node involvement was as high as 60–90% 1, 3, 18, 19, 20, 21, and Shi et al. reported 51.3% cases of IMPC presented with AJCC stage III 16. The relatively lower percentage in our study may be the result of effectiveness of breast cancer screening and improvement in early diagnosis. Due to the aggressive clinical presentations, more IMPC patients underwent mastectomy in our study. But many IMPC patients who were not suitable for BCS might go for reconstruction, resulting in an obvious higher rate of breast reconstruction compared with IDC. It reflected the improvement in locoregional treatment modality as well as the increasing aesthetic demands.

Due to the aggressive and distinct clinicopathologic characteristics of IMPC, the survival outcomes of IMPC were generally accepted to be worse than those of IDC 10, 11. But in fact, the survival outcome of IMPC reported from the literature was inconsistent. Although a case–control study showed IMPC histotype did not add any independent information to the risk of locoregional recurrence (LRR) (*P* = 0.48) 12, most studies acknowledged that IMPC had a higher LRR risk than IDC 13, 14. Our previous study also indicated that IMPC had an inferior LRR‐free survival (LRRFS) rate compared with triple negative breast cancer (TNBC), which was the most aggressive subtype of IDC 15. Some research with small sample size demonstrated a lower 5‐year BCSS and recurrence‐free survival rate (75.9% and 67.1%, respectively) than patients with IDC (89.5% and 84.5%, respectively) 16, but many large retrospective studies have shown that the OS of IMPC is not inferior to that of IDC 3, 4, 13. Prior to our study, Chen et al. evaluated 636 IMPC patients diagnosed between 2001 and 2008 in SEER database and indicated that IMPC had a BCSS and OS comparable to those of IDC in spite of its high propensity for lymph node metastasis 4. But this study included about 4% stage IV cases. As for the great difference in the number of cases (636 IMPCs vs. 297735 IDCs) and the absence of case‐matched analysis, query might exist that the significant differences between the cohorts of IMPC and IDC could be the reasons of the differences observed in terms of prognosis. Although Vingiani indicated that IMPC had a similar survival outcome with IDC through a case–control study, a relatively small sample size (49 IMPCs vs. 98 IDCs) might also compromise the efficacy 12. Our study included 984 IMPC cases in stage I‐III with a relatively adequate follow‐up period. It confirmed that IMPC had a better survival outcome than IDC through both the comparison based on the large population database and a 1:1 matched case–control analysis as well. As 39 IMPCs in stage III (17.6%) lost in the completely matched case–control analysis, there might be some bias in the analysis. Another partly matched case–control analysis was further conducted with at least nine variables matched including year of diagnosis, histologic grade, tumor size, node status, ER status, and so on, which were strongly related with the survival of IMPC. It drew the same conclusion that IMPC had a better survival outcome than IDC through multivariate analysis.

Then, it comes the question that whether LRR impairs long‐term survival. In IDCs, LRR may be responsible for an increase in distant metastasis and disease‐specific mortality in patients who undergo BCS and receive RT 22. In the meantime, the HR for disease‐specific death among women undergoing mastectomy with an LRR 0.5–1 year after diagnosis was 6.67 (95% CI: 3.71–11.99) when compared with women who did not experience LRR 23. But the situation may be quite different in IMPC. A retrospective multicenter study in Korea found IMPC had a higher propensity for LRR than randomly matched IDC (*P* = 0.03) without increasing the probability of distant metastasis (*P* = 0.52) or compromising the OS (*P* = 0.67) rate 14. Our previous study compared IMPC and TNBC and found that IMPC had a significantly lower 5y‐LRRFS rate than TNBC (*P* < 0.001) but had a similar 5y‐OS (*P* = 0.475). A tendency of lower 5y‐distant metastasis‐free survival rate was observed in TNBC compared with in IMPC, in node positive cases (*P* = 0.053) and in node negative cases (*P* = 0.052) 15. It seemed adequate local treatment may be of much help for IMPC. The mechanism warrants further research. Modification of the locoregional treatment modality might be needed in this pathologic subtype of breast cancer.

According to the subgroup analysis based on case–control analysis, there was no statistically significant difference in BCSS and OS between IMPC and IDC in AJCC stage I and histologic grade I. Probably due to relatively good survival outcome of IDC in these cases, the survival advantage of IMPC might not be so apparent. But in more advanced stages and histologic grades, the survival advantage of IMPC became so apparent.

Our study found that the rate of ER and PR positivity was 88.0% and 75.7% respectively, significantly higher than that of IDC, which was in accord with most literature 2, 3, 9, 12, 24. The comparable survival outcome of IMPC was usually attributed to the high rate of ER positivity 3, 4. But according to the subgroup analysis based on case–control analysis, IMPC exhibited better BCSS and OS in each ER and PR status. So there may be important mechanisms underlying for the improved survival of IMPC rather than higher proportion of hormone receptor positivity.

A recent expression profile study of various special types of breast cancer suggested a unique molecular genetic disease profile for IMPC 25. Other molecular genetic studies demonstrated that IMPC had distinct molecular genetic profiles, supporting the contention that they constituted a distinct pathological entity 26, 27. And IMPCs were not defined by highly recurrent mutations in many genes tested in breast cancer 28. Some genetic events may play an important role in its unique pathologic features and clinical presentations. Correlative studies should focus on this subset of patients to elucidate the genetic and biologic differences that contribute to the better overall survival in spite of aggressive LRR.

We recognize several limitations of this study. First, this study was a retrospective study and the intrinsic defects existed in any retrospective study despite we had a large sample size. And the study was based on a single large population database. Second, we were unable to attain information on HER2 status of tumors before 2010, and we were unable to attain the information of endocrine therapy either, which was the important treatment for IMPC with high positivity of ER/PR, their effects on survival could not be evaluated. Third, as SEER database did not provide the information of LRR, which was an important characteristic of IMPC, this survival outcome could not be analyzed further. And lastly, the database did not specify the proportion of the IMPC component in each lesion. However, no consensus has yet been reached on the proportion of the IMPC component in a tumor that is required for its pathologic diagnosis. There was no statistically significant difference between pure IMPC and mixed IMPC, in terms of mean age of the patients, mean tumor size, presence of high‐grade tumor, axillary lymph node metastasis, local recurrence rate, or overall survival 2.

## Conclusion

IMPC has a better long‐term survival outcome with statistical significance compared with IDC in BCSS and OS in spite of its highly aggressive clinical presentation, such as larger tumor size, higher proclivity for lymph node metastasis, and advanced tumor stage. The genetic profiling and biologic differences that contribute to the better survival warrant further research.

## Conflicts of Interest

None declared.
